# Adsorption of nanoparticles suspended in a drop on a leaf surface of *Perilla frutescens* and their infiltration through stomatal pathway

**DOI:** 10.1038/s41598-021-91073-x

**Published:** 2021-06-02

**Authors:** Nami Ha, Eunseok Seo, Seonghan Kim, Sang Joon Lee

**Affiliations:** 1 Department of Mechanical Engineering, Center of Biofluid and Biomimic Research, Pohang University of Science and Technology (POSTECH), 77, Cheongam-ro, Nam-gu, Pohang-si, Gyeongsangbuk-do Republic of Korea; 2 Division of Mechanical Engineering for Creative Emerging Technologies, Center of Biofluid and Biomimic Research, Pohang University of Science and Technology (POSTECH), 77, Cheongam-ro, Nam-gu, Pohang-si, Gyeongsangbuk-do Republic of Korea; 3 Department of Mechanical Engineering, Pohang University of Science and Technology (POSTECH), 77, Cheongam-ro, Nam-gu, Pohang-si, Gyeongsangbuk-do Republic of Korea

**Keywords:** Imaging, Stomata, Natural hazards, Nanotoxicology, Environmental, health and safety issues

## Abstract

Particulate matter (PM) has become a severe environmental issue, and ultrafine PM particles such as PM_2.5_ or PM_1_ can cause various complications and respiratory diseases to human beings. In particular, heavy metals contained in PM particles can contaminate edible plants; for example, plant leaves are exposed to PM particle-laden raindrops. The contaminated edible plants can injure the human health by ingestion, so a detailed understanding on the accumulation of PM particles inside edible plants is essential. In this study, we investigate the infiltration of PM particles in plant tissues with a hypothesis that ultrafine PM particles are absorbed through stomatal pathways. As an edible test plant, *Perilla frutescens* is selected. Drops of gold nanoparticle (AuNP) suspension are deposited on a leaf of *P. frutescens* to simulate the scenario where PM particle-laden raindrops fall on patulous stomata of the test plant. To examine AuNP adsorption on the *P. frutescens* foliar surface and diffusional AuNP absorption through stomatal apertures, we investigate three physical dynamics of AuNPs suspended in a sessile drop: sedimentation, evaporation-driven convective flow, and shrinkage of the drop interface. Quantitative information on the 3D spatial distribution of AuNPs in plant tissues was measured by X-ray imaging and two-photon excitation microscopy.

## Introduction

Particulate matter (PM) particles are complex mixtures of hazardous solid and liquid aerosols suspended in the air, and they cause severe environmental issues. PM particles are classified into PM_10_, PM_2.5_, and PM_1_ based on the upper limit size of aerosols at 10, 2.5, and 1 μm, respectively. Ultrafine PM particles such as PM_2.5_ or PM_1_ can cause complex complications and respiratory diseases^1^. The effects of PM_2.5_ particles on mortality and chronic cardiovascular diseases, such as heart failure and diabetes, were recently reported^2^. In addition, PM_2.5_ particles can cause lung cancer and premature death^3^. Airborne PM particles can be exposed to humans by two main routes: direct breathing^3^ and food consumption^4^. Through direct breathing, PM particles are deposited on the respiratory system by inhalation, and they permeate into blood vessels^3^. Food consumption is another important infiltration pathway of PM particles, and uptake of edible plants is one of the main infiltration routes^4^. Therefore, an investigation on the dynamical motions and accumulation of PM particles in edible plants is important^5^.

Given that atmospheric PM particles can be adsorbed to the plant surfaces and into the plant tissues, some plant species have been utilized for air purifying or biomonitoring purposes^6^. For example, *Tillandsia usneoides*, used for purifying indoor air, has densely distributed trichomes on its surface and captures both incense and solid PM particles effectively under the flow condition due to the increased surface area by trichome arrays^7^. The settling trajectories of PM particles to the surface of *Perilla frutescens* were recently studied by digital holographic microscopy to quantify the sedimentation velocity of PM particles by gravitational forces and electrostatic forces^8^. However, compared with the adsorption of PM particles on the leaf surface, the absorption of PM particles into the plants is poorly understood.

The absorption of contaminants into edible plants is driven by foliar and root uptake^9–11^. However, the foliar uptake and transfer mechanism has been less explored in comparison with soil–root transfer via water in the hydroponic system^12,13^. Previous studies revealed that the foliar uptake of PM particles occurs through stomatal and cuticular entrances^14^. To cross the entrances, size exclusion limits exist^15^. Previous work hypothesized that only nanoparticles (NPs) much smaller than 100 nm can cross the cuticular entrance^16^. For this reason, PM particles with submicro/micro sizes are mainly absorbed through stomatal pathways^9^. The foliar uptake of PM particles usually occurs in the industrial regions^17^ or kitchen gardens^18^.

Another infiltration pathway is to absorb PM particles suspended in liquid by spraying^19,20^, fog/cloud droplet deposition^21^, raindrop splashing^22,23^, or resuspension of dust^24^. These phenomena are secondarily or naturally generated based on PM size, and they belong to wet deposition, which results from the inclusion of the atmospheric particles and gases into water drops^25^. For instance, residual PM particle-laden drops are usually observed on foliar surfaces after rainfall, which can contaminate edible plants and disrupt their metabolism. Moreover, wet deposition increases the residence time, retention amount, and influx of PM particles to edible plants compared with dry deposition, which is mainly driven by gravitational sedimentation and weak electrostatic force near the vegetation surface^26,27^. As a result of the short period of precipitation and sudden temperature decline, dry-deposited PM particles are hydrated and infiltrate the edible plants^26^. Edible plants are even more susceptible if epicuticular waxes are removed due to repetitive drop impacts^28^. Stomatal uptake of PM particles is limited by the stomatal aperture size, stomatal number density, and stomatal opening cycle^14^. Nonetheless, the absorption of PM particles suspended in raindrops into leaves of edible plants remains unclear.

In this study, wet deposition and absorption of ultrafine PM particles suspended in sessile drops into an edible plant, *Perilla frutescens*, was investigated by assuming infiltration through stomatal pathways. As raindrops are mixed electrolytes that contain various ions such as sodium, potassium, magnesium and chloride etc.^29^, phosphate buffered saline (PBS) was selected as an experimental salt fee solution. Although the pH of actual raindrops is smaller than PBS or water as atmospheric carbon dioxide is dissolved in the drops, effects of acidification and variation in the colloidal stability of particles were not considered in this study to focus on the geometrical and dynamic effects of particles. In addition, most of ultrafine PM particles, such as PM_1_ or PM_2.5_ particles of urban road dusts, have submicron-size and some of them have even size smaller than 100 nm^24,30^. The main components of road dusts, for instance, Arizona road dust, are silica, aluminum oxide, calcium oxide, potassium oxide, sodium oxide, iron oxide, magnesium oxide and titanium oxide, and their average size and density were previously reported as 0.3 μm^31^ and 2.65 g/cm^3^^32^, respectively. Referring to this information, spherical gold nanoparticles (AuNPs) stabilized in PBS were selected as an experimental model of this study because they would have similarity with the Arizona road dust (A1 dust) in the geometrical size and dynamic behaviors such as wet deposition behavior and stomatal infiltration. The size of AuNP used in this study is about 100 nm in diameter, and their surface has no chemical coating to minimize the effects of different wetting properties of particles, such as lipophilicity and hydrophilicity^14,33^. AuNPs have been used as experimental metal model to investigate foliar uptake of NPs due to their biocompatibility or chemical stability^13,32^. In addition, they are also suitable for visualization researches applying advanced imaging techniques, adapted in this study, such as X-ray imaging and two-photon excitation microscopy imaging (TPEM)^34^. The presence of AuNPs in the vacant region below the stomata was already reported^14,15,35^, but studies on physical understandings of wet deposition and absorption of NPs laden in suspension drops remain insufficient. On the basis of the three physical phenomena of sedimentation, evaporation-driven convective flow, and shrinkage of drop interface, we explored the transport of AuNPs suspended in a sessile drop to the stomatal apertures and diffusional AuNP absorption through the apertures, and discussed the similarity with the transport of A1 dusts. The present results would be helpful for understanding the toxicological effects of ultrafine PM particles on edible plants by simulating the scenario where stomata are covered with raindrops containing PM particles. Moreover, X-ray imaging and TPEM techniques employed to investigate AuNP absorption in plant tissues are helpful for analyzing the dynamic behaviors of PM particles accumulated in living organisms, such as plant species. Finally, we discussed the possible absorption process of NP-laden drop through a stomatal opening, and the subsequent translocation routes of NPs.

## Results and discussion

### Adsorption of AuNPs on the abaxial surface by wet deposition

**Figure 1:**
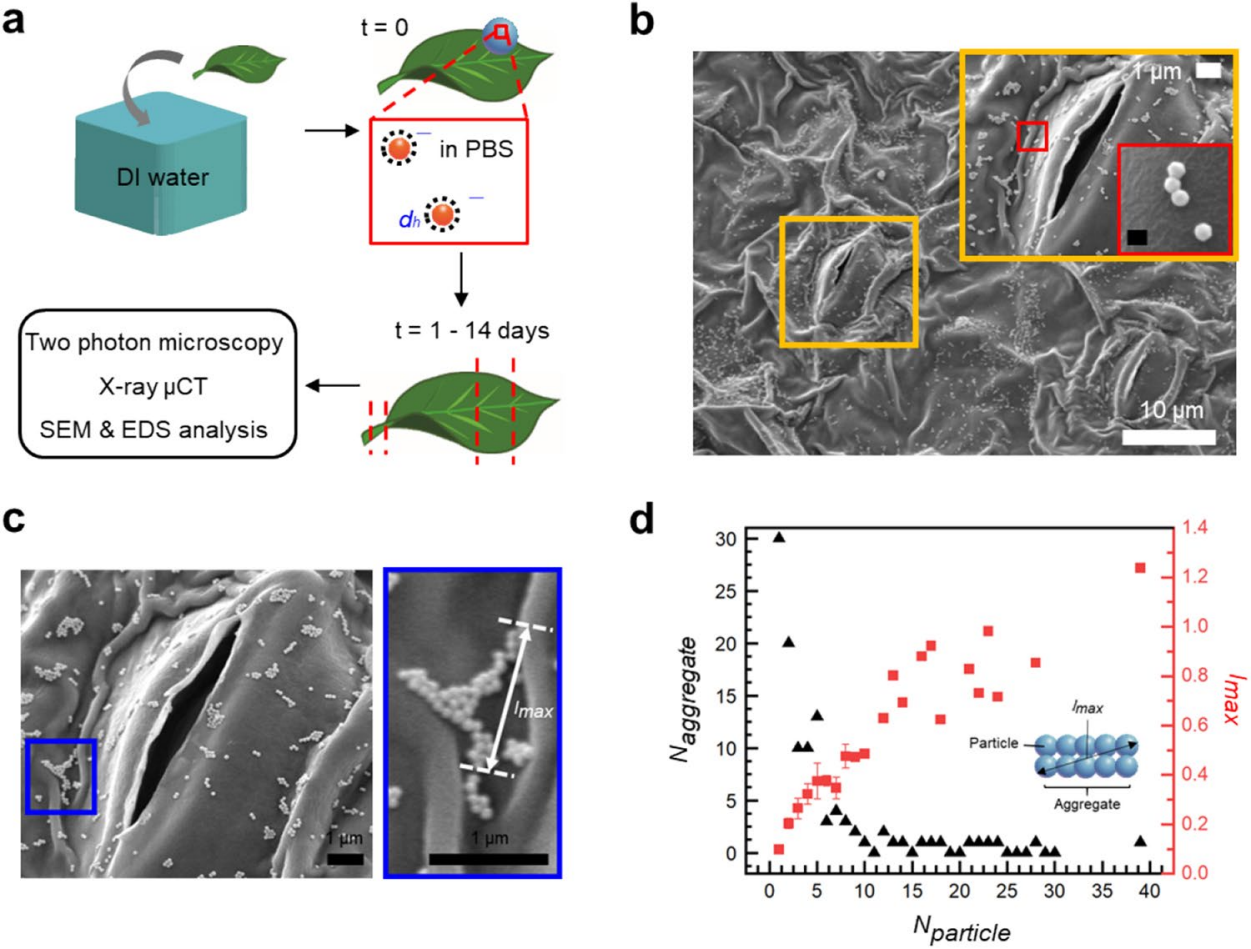
Adsorption of AuNPs on the abaxial surface of *Perilla frutescens* by drop deposition method. (**a**) Experimental schematics of exposing *Perilla frutescens* leaf to gold nanoparticle (AuNP) suspension. After washing the leaf, 30 μl drops of AuNP suspension are deposited on the abaxial side of the leaf. After exposure for 1–14 days, the leaf and stem are extracted to investigate the absorption of AuNPs through the stomatal pathway by using two-photon excitation microscopy (TPEM) imaging technique, X-ray micro-computed tomography (μCT), and SEM and EDS analysis. (**b**) Typical SEM image showing the adsorption of AuNPs on the abaxial side of *P. frutescens* leaf. Inset shows the magnified stoma in the yellow box. Aggregates of AuNPs are shown in the red box. Scale bar is 100 nm. (**c**) SEM images showing the magnified stoma (left) and maximum length scale (*l*_*max*_) of AuNPs (right). (**d**) Variations in the number of aggregates (*N*_*aggregate*_) (black triangles) and maximum length scale (*l*_*max*_) (red squares) with respect to the number of particles per aggregate (*N*_*particle*_) in the region near the stoma in (**c**). Error bars indicate standard deviations (SD).

The deposition of AuNPs on the abaxial surface of *P. frutescens* was experimentally investigated (Fig. 1). SEM images show the spatial distributions of AuNPs near the stomata after the evaporation of drops (Fig. 1b). The stomatal aperture indicated in the yellow box was open, and the AuNPs were distributed around the guard cells. This distribution of AuNPs near the stomatal openings suggested that AuNPs might be diffused into the voids. Most AuNPs observed in the inset were single particles or small-scale aggregates composed of 2–5 particles due to the well-dispersed AuNPs by ultrasonication, but a few large-size aggregates comprising 10–30 particles were also observed (Fig. 1c). Due to the aggregation, the maximum length scale (*l*_*max*_) of several AuNP clusters was larger than 100 nm, and the biggest AuNP aggregates (*N*_*particle*_ = 38) was ~ 1.25 µm, implying that some AuNP aggregates might act as microparticles in a sessile drop (Fig. 1d). After the observation of AuNP adsorption on the abaxial surface of *P. frutescens*, we analyzed what happens to AuNPs in a sessile drop in association with AuNP absorption through stomatal pathways.

### Physical dynamics of AuNPs in a sessile drop

Subsequently, we analyzed dynamic behaviors of AuNPs in a sessile drop before they were adsorbed on the surface of *P. frutescens*. Impingement of particle-laden drops on a soft surface is a complex problem, including oscillatory vibration of the leaf^28^, vortex shedding near the boundary^36^, and elastic deformation of the foliar surface^37^. Thus, we simplified the problem in this study and focused on the dynamic behaviors of AuNPs in static drops. As the size of raindrops generally ranges from 0.5 to 3 mm, AuNP-laden drops within the range were used in this study^38^. Using the sessile drops, we investigated AuNP sedimentation, evaporation-driven internal flow in the AuNP-laden sessile drop, and shrinkage of drop interface, as well as how these phenomena are related to AuNP infiltration.

The sedimentation velocity (*v*_*sed*_) of individual AuNP was estimated by balancing the gravitational ($f_{g}$), buoyant ($f_{b}$) and viscous forces ($f_{v}$). For a spherical colloid, viscous drag force is given as $f_{v} = 6\pi \eta Rv$ by Stokes law, and the resultant *v*_*sed*_ is

1 $$v_{sed} = \frac{1}{18}\frac{{d_{p}^{2} (\rho_{p} - \rho_{f} )g}}{\eta } = \frac{1}{18}\left( {\frac{{\rho_{p} }}{{\rho_{f} }} - 1} \right)\frac{{gd_{p}^{2} }}{\eta }$$

when the net force $f_{net}$ satisfies the condition of $f_{net} = f_{g} - f_{b} - f_{v} = 0$. The densities of AuNPs ($\rho_{p}$) and PBS solution ($\rho_{f}$) are 19.3 and 1 g/cm^3^, respectively. The dynamic viscosity of PBS solution is ~ 10^–3^ Pa∙s, similar to that of water. Accordingly, the sedimentation velocity *v*_*sed*_ of AuNP of diameter $d_{p}$ = 100 nm is 101.7 nm/s ~ O (10^–1^) µm/s. Thus, the settling distance (*l*_*settl*_) of a single AuNP in the bulk PBS solution is approximately 366.12 µm in 1 h. The similarity in the theoretical *v*_*sed*_ values between AuNPs and road dust particles was investigated using Eq. (1) under a condition where particles are suspended in liquid with density of $\rho_{f}$ ~ 1 g/cm^3^. The *v*_*sed*_ and *l*_*settl*_ were estimated by considering the density and size of road dust particles and AuNPs, obtained from the captured SEM images and measured hydrodynamic diameter (that will be explained in the next section). As a result, the *v*_*sed*_ values of AuNPs and road dusts are similar in the range of O (10^–1^–10^0^) µm/s (Table S1, Fig. S1).

**Figure 2:**
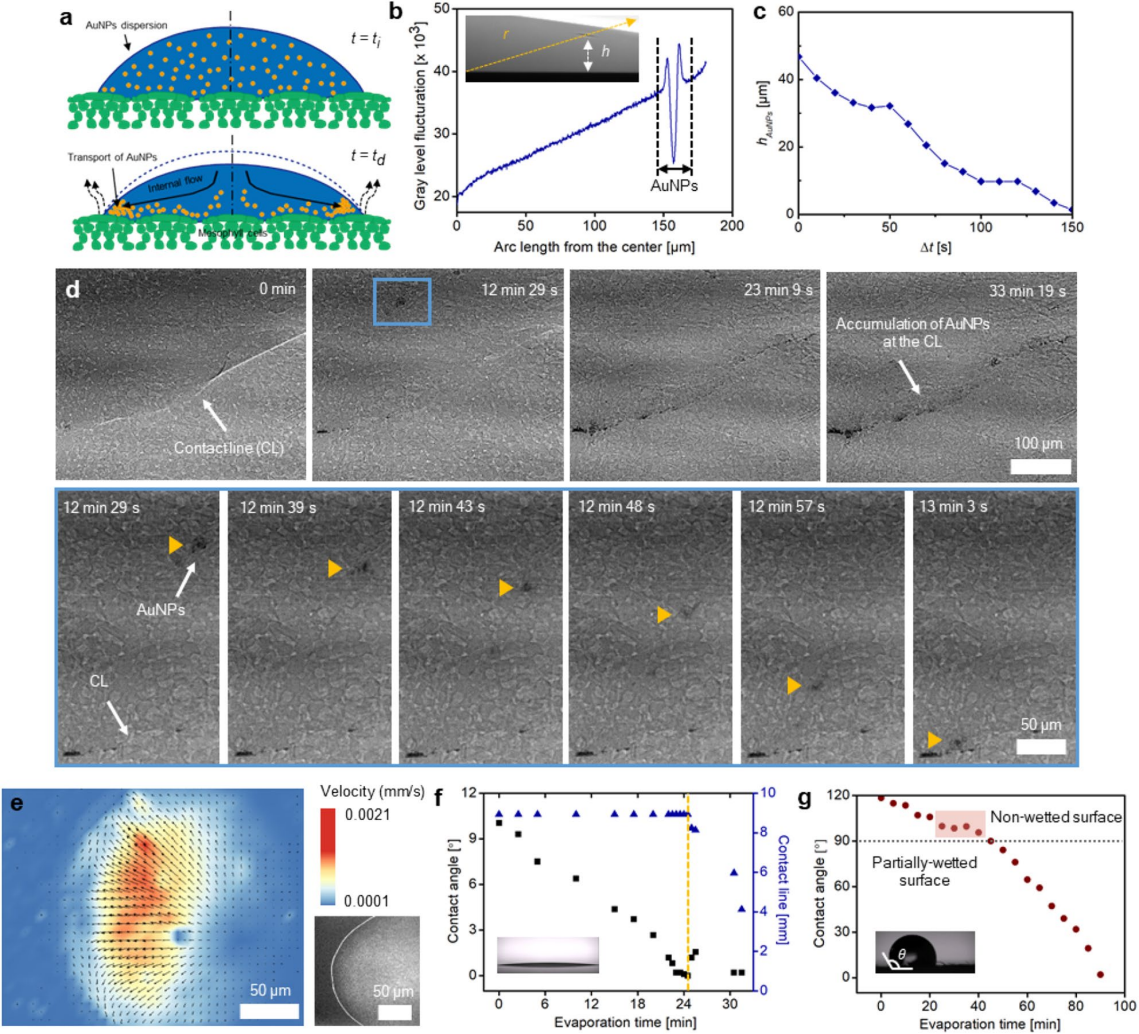
Transport of AuNPs by the convective internal flow in a sessile drop. (**a**) Schematics showing the dynamic behaviors of AuNPs moving toward the contact lines (CL) of a drop on the leaf surface by evaporation-driven internal flow. At the initial time (*t* = *t*_*i*_), AuNPs are uniformly distributed in the sessile drop. As the drop evaporates, AuNPs are translocated to the regions near the leaf surface. (**b**,**c**) Dynamical variations in AuNPs when a drop evaporates on a glass substrate. (**b**) Gray level fluctuations indicating the light intensity profile show the position of AuNP clusters along a yellow line. Inset is a contrast-enhanced X-ray image showing the side view of the drop. (**c**) Temporal variation in the height of AuNP clusters (*h*_*AuNPs*_). The gradual decrease in *h*_*AuNPs*_ shows the transport of AuNPs toward the glass surface. (**d**) Sequential X-ray images show the top view of AuNP clusters gathering toward the CL region due to evaporation-driven internal flow. Accordingly, stomata positioned near the CL are easily infiltrated by wicking of AuNP suspension. Magnified images in blue box show temporal variations in the position of AuNP clusters. (**e**) Velocity field of the flow in the sessile drop deposited on a glass substrate, measured by applying a PIV technique from the top view. (**f**,**g**) Temporal variations in contact angle (CA) of a sessile drop on (**f**) a flat glass substrate and (**g**) the abaxial surface of *P. frutescens* (*n* = 1). Insets show the side view of a 2 μl sessile drop and $\theta$ indicates CA.

In addition to the passive sedimentation of AuNPs, the movement of AuNPs by the evaporation-driven convective flow in a sessile drop was explored (Fig. 2a). Contrast-enhanced X-ray imaging allows individual AuNPs in a drop to be tracked (Fig. 2b). Sequential contrast-enhanced X-ray images of a drop on the surface of *P. frutescens* showed that AuNPs moved toward the region of contact lines (CL), which indicated that another driving force functioned as well as sedimentation (Fig. 2c). As shown in the top view of Fig. 2d, AuNPs spread along the outer radial direction, and they accumulated in the region near the CL as the drop evaporated for 33 min. The residual particles in a coffee-ring pattern were attributed to high evaporation rates in the region near the drop CL, as explained by Deegan et al.^39^.

To support our claim that evaporation-driven internal convective flow is dominant compared with sedimentation during deposition process of AuNPs and dust particles, the velocity field in a sessile drop deposited on a flat glass substrate was measured by using a particle imaging velocimetry (PIV) velocity field measurement technique (Fig. 2e). To reduce the thermal effect of laser on drop evaporation, laser was irradiated at low light intensity^40^. Polystyrene particles (PSP) of 1 µm in diameter were selected as tracing fluorescent particles to ensure the similar theoretical *v*_*sed*_ value with those of AuNPs and ultrafine A1 dust particles. The *v*_*sed*_ value of a single PSP was estimated as *v*_*sed*_ ~ 0.03 µm/s or 100 µm/h from Eq. (1), implying that aggregates of PSP would show *v*_*sed*_ ~ O (10^–2^–10^–1^) µm/s. The measured velocity fields in the top view exhibited forward and backward flows due to recirculation of internal flow (Fig. 2e). The velocity scale of the forward flows was higher than that of the backward flows. The maximum forward flow velocity was 2 µm/s ~ O (10^0^) µm/s or 7.2 mm/h. Thus, the effect of internal convective flow for PSP was dominant compared with that of sedimentation.

Theoretically, evaporation-driven internal convective flow resulted from non-uniform evaporative mass flux (*J*), which is a function of radial length (*r*) and contact angle (CA) ($\theta$) under constant thermal condition. According to the simplified Deegan’s model for a partially wetted surface,

2 $$J(r) = J_{o} (\theta )\left[ {1 - \left( \frac{r}{R} \right)^{2} } \right]^{{ - \left( {0.5 - \frac{\theta }{\pi }} \right)}}$$

with $J_{o} (\theta ) = [D(c_{sat} - c_{\infty } )/R](0.27\theta^{2} + 1.3)(0.638 - 0.224(\theta - \pi /4)^{2} )$ where *R*, *D*, *c*_*sat*_, and $c_{\infty }$ denote the wetted radius of the drop, the diffusion coefficient of liquid vapor into the surroundings, and the saturated and ambient vapor concentrations, respectively^41^. The high evaporation rate at a large radius induces a temperature gradient in the drop and generates thermocapillary (Marangoni) flow. The downward flow in the center region on the partially wetted surface would make AuNPs attach to the leaf surface, especially to the CL regions^42^. However, the drop on the surface of *P. frutescens* was under non-wetted state due to the presence of microstructures at the initial stage (Fig. 2g). It became a partially wetted state after 40 min due to the transition from Cassie state to Wenzel state. During the initial stage of Cassie state, the central upward flow dominantly transferred the AuNPs, leading to AuNP accumulation near the central regions of the deposited drop^41^. Central downward flow was generated after the wettability transition to Wenzel state, which would dominate AuNP transport toward the CL, as observed in the captured X-ray images (Fig. 2d).

In addition to evaporation-driven internal flow, the shrinkage of the sessile drop interface may affect the adsorption behavior on the *P. frutescens* surface. Given that the foliar surface has complicated structures, the exact CL cannot be easily discriminated. For this reason, we compared temporal variations of CA and CL values on a flat glass surface (Fig. 2f). The CA on the glass decreased, while the CL was pinned up to the initial 24 min. Thereafter, the CL receded to reduce the surface energy and the CA increased for a short period. This stick–slip behavior, pinning, and depinning of CL on the glass during drop evaporation made AuNPs accumulated in the CL regions^41^. Based on these results on the glass surface, we speculated that this stick–slip behavior would also be generated on the surface of *P. frutescens*. The CA value on the *P. frutescens* surface decreased; however, in a certain period (20–40 min), the decrease in CA was delayed as indicated in the red box (Fig. 2g). This delay might arise from stick–slip behavior, although the exact CL cannot be easily distinguished due to the presence of various microstructures on the *P. frutescens* surface, such as trichomes and grooves. In addition, a thin film of AuNP suspension may experience the stick–slip behavior after the CA becomes nearly zero. Thus, most AuNPs on the *P. frutescens* surface would remain in the regions near the CL, increasing the adsorption of AuNPs to stomata and guard cells distributed around the CL.

### Absorption of AuNPs through the stomatal pathway in P. frutescens

**Figure 3:**
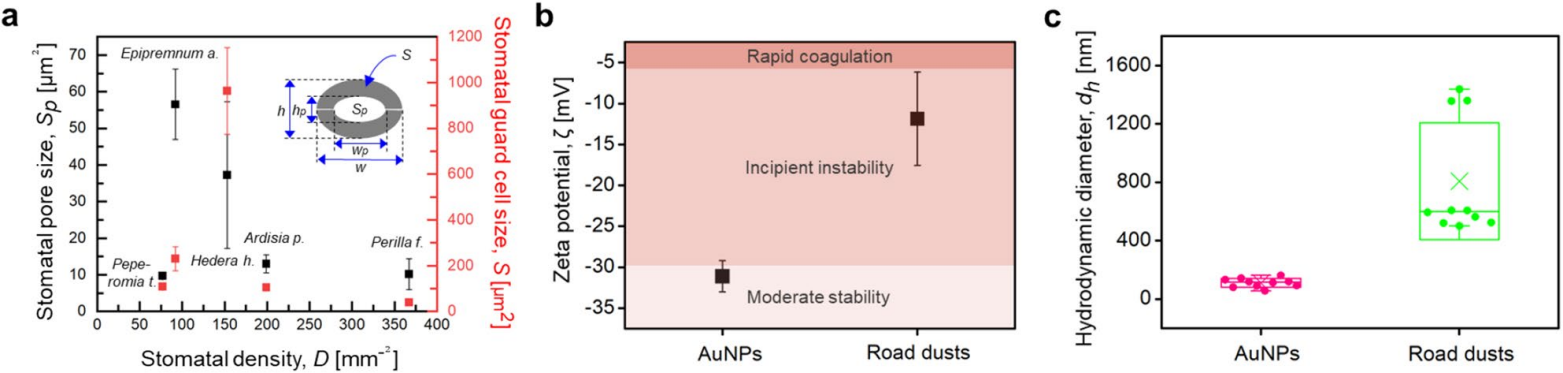
Geometrical/chemical features of stomata and AuNPs related to the absorption of PM particles through stomatal openings. (**a**) Comparison of stomatal guard cell size (*S*) and stomatal pore size (*S*_*p*_) versus stomatal number density (*D*) of *P. frutescens* and four air-purifying plant species, *Peperomia t.*, *Epipremnum a.*, *Hedera h.*, and *Ardisia p.* An inset describes the stomatal dimensions. By assuming elliptical-shaped stomata, *S*_*p*_, *h*_*p*_, and *w*_*p*_ indicate the size, minor axis, and major axis of the elliptical stomatal pore, respectively, while *S*, *h*, and *w* represent those of stomatal guard cells. Stomatal number density (*D*) indicates the number of stomata per unit area. (**b**) Zeta potential (*ζ*) and (**c**) hydrodynamic diameter (*d*_*h*_) of AuNPs and road dusts. The *ζ* values of AuNPs and road dusts support that AuNPs are moderately stable, while road dusts (solid PM_2.5_ particles) are incipiently instable. The *d*_*h*_ values of AuNPs are much smaller than those of road dusts. Error bars indicate standard deviations (SD).

On the basis of the hypothesis that AuNPs are absorbed through stomatal pathways, the geometrical parameters of *P. frutescens*, such as stomatal guard cell size (*S*), stomatal number density (*D*), and stomatal pore size (*S*_*p*_), were compared with those of four air-purifying plants, *Peperomia tetragona*, *Epipremnum aureum*, *Hedera helix*, and *Ardisia pusilla*, whose species are known to remove air contaminants such as volatile organic compounds (VOCs) of benzene^43^, toluene^44^, and formaldehyde^45^ (Fig. 3a). By assuming elliptical-shaped stomata, the guard cell size and stomatal pore size can be expressed as *S* = $\pi$ *wh* and *S*_*p*_ = $\pi$ *w*_*p*_*h*_*p*_. The average *S* and *S*_*p*_ values of the tested *P. frutescens* sample were 36.4 ± 7.8 µm^2^ and 8.3 ± 4.3 µm^2^, respectively, implying that the stomatal pore size was relatively small among the five plant species but sufficient for AuNPs to enter the apertures of *P. frutescens*. Moreover, the stomatal number density of *P. frutescens* was 367 mm^−2^, which was the highest among the five plant species (Table 1). As a result, *S*_*p*_ of *P. frutescens* was approximately 328 times larger than the surface area of spherical AuNPs of 100 nm in diameter (*S*_*AuNPs*_). For the adaxial leaf of 1 mm^2^ area, the total *S*_*p*_ was 3780 µm^2^ and 10^5^ times larger than *S*_*AuNPs*_ when all the stomata were open at the maximum.

**Table 1:** Dimensions of stomata on the adaxial surface of *Perilla frutescens*.

Symbol	Definition	Size	SD	*n*
S	Stomatal guard cell size	36.4 μm^2^	7.8 μm^2^	5
*S*_*p*_	Stomatal pore size	8.3 μm^2^	4.3 μm^2^	5
*D*	Stomatal number density	367 mm^−2^	–	1
*h*	Minor axis of stomatal guard cell	4.3 μm	1.1 μm	5
*w*	Major axis of stomatal guard cell	10.8 μm	1.1 μm	5
*h*_*p*_	Minor axis of stomatal pore	1.4 μm	0.6 μm	5
*w*_*p*_	Major axis of stomatal pore	7.3 μm	1.0 μm	5

In addition, AuNP aggregation would alter the possibility of absorption through stomatal pores, meaning that individual AuNPs would receive less resistance than large-scale aggregates for entering the stomatal apertures. We investigated this property associated with particular attraction of AuNPs by comparing zeta potential (*ζ*) with that of solid road dusts used as commercial PM_2.5_ particles (Fig. 3b). *ζ* values of AuNPs and road dusts were − 31.1 ± 1.9 and − 11.9 ± 5.7 mV, respectively, which indicated that both AuNPs and road dust particles showed the electronegativity, but AuNPs were in the moderately stable state, while road dusts were in the incipiently instable state. Thus, for the particles suspended in sessile drops, AuNPs might enter the stomatal apertures easily than the solid dusts, because the dusts have a high propensity to aggregate. Moreover, the hydrodynamic diameters (*d*_*h*_) of AuNPs and road dusts were 111 ± 31 and 807 ± 401 nm, respectively, which indicated that road dusts would have a lower probability of entering through stomatal pathways (Fig. 3c). The measured *d*_*h*_ of road dusts was in the similar range with those measured in previous studies (Table S2). Further, 61% of A1 dust particles in the number distribution belongs to submicron in their size, and consequently, most of A1 dust particles could be inferred as nanoparticles (Fig. S2).

Concerning the mechanism of NP absorption laden in a drop, the gradients of chemical potentials of AuNPs across the stomatal aperture would result in the diffusion of AuNPs from the surface to the internal voids of sponge mesophyll cells. We could simplify this problem as 1D diffusion by adopting Fick’s first law. In general, the diffusion coefficient (*D*) of colloidal particles is scaled as *D* ~ 10^–11^ m^2^/s, and the concentration of AuNP suspension is 3.8 × 10^9^ particles/ml (*c*_*1*_). By assuming that no AuNPs (*c*_*2*_ = 0) in plant tissues existed at the initial time, the theoretical diffusion flux (*J*_*diff*_) across the characteristic length scale ($\Delta x$) of 10 µm would be $J_{diff}$ $\cong$ 3.8 × 10^3^ [particles/mm^2^ s] based on Fick’s first law.

3 $$J_{diff} = - D\left( {\frac{\partial c}{{\partial x}}} \right)$$

Thus, the initial *J*_*diff*_ through the stomatal aperture of *S*_*p*_ = 10.3 μm^2^ would be *J*_*diff *_$\cong$ 3.9 × 10^–2^ [particles/stomata $\cdot$ s] $\cong$ 140 [particles/stomata $\cdot$ h] across the internal voids of 10 µm in depth. Considering the stomatal number density of 367 [mm^−2^], 51,380 particles might diffuse into the depth of 10 µm through stomatal pathways per abaxial surface of 1 mm^2^ in 1 h. The concentration of AuNPs near the surface would rapidly increase due to the transport of AuNPs by evaporation-driven convective flow in sessile drops. This phenomenon would enhance the local concentration of AuNPs in the regions around the stomata, especially near the CL. Therefore, the diffusional flux $J_{diff}$ would further increase as the initial local concentration of AuNPs (c_o_) increases, as described in Fick’s second law.

4 $$c(x) = c_{o} \left( {1 - \left( {\frac{x}{{\sqrt {Dt\pi } }}} \right)} \right)$$

**Figure 4:**
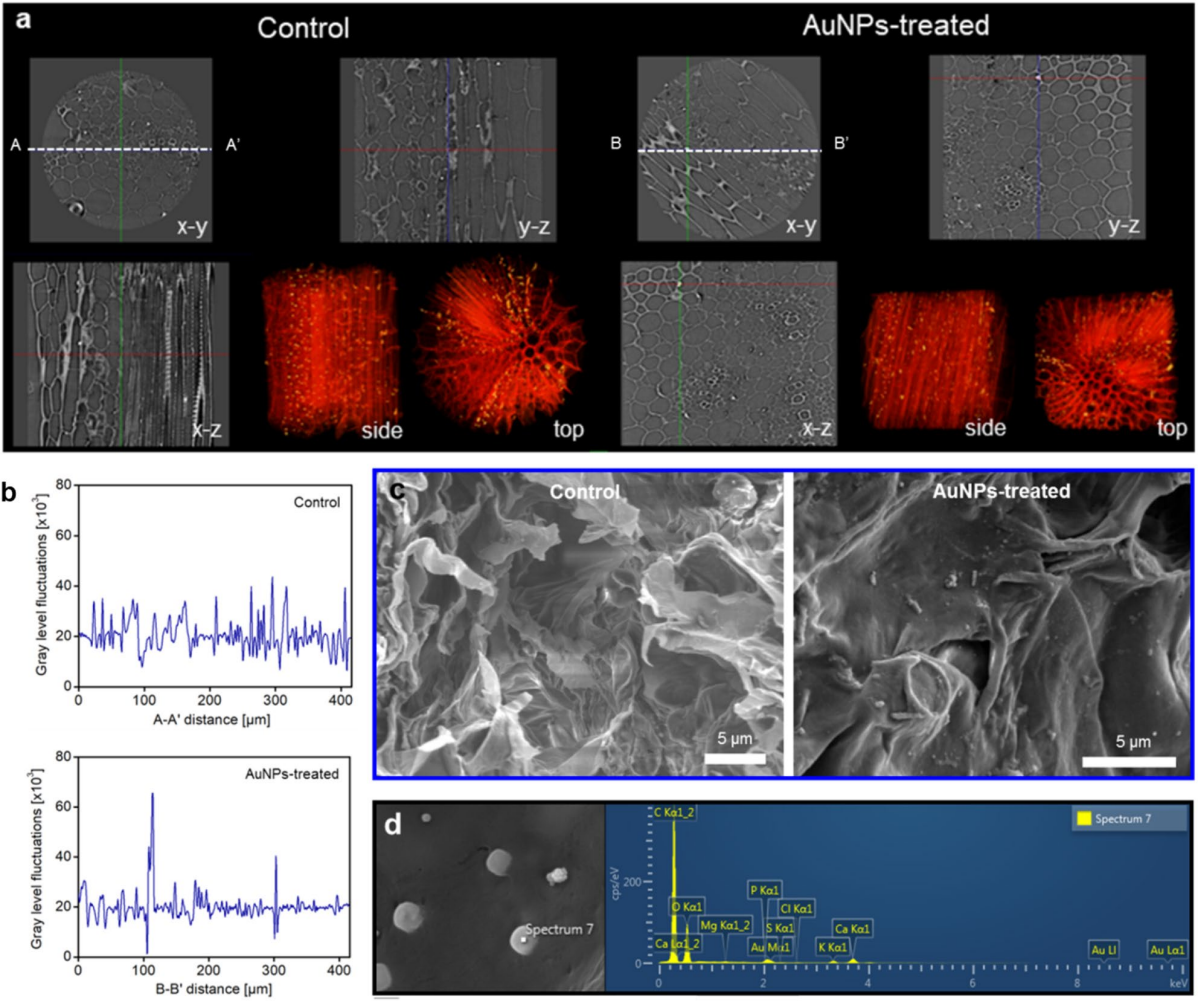
Visualization of AuNPs absorbed into on the surface of *Perilla frutescens* through stomatal pores. (**a**) 3D X-ray micro-CT images of the control (left) and AuNP-treated (right) *P. frutescens*. Cross-sectional images in the x–y, y–z, and x–z planes show the internal morphological structure of *P. frutescens.* The reconstructed 3D images show morphological structures of mesophyll cells and veins of *P. frutescens*. In contrast to the control *P. frutescens,* the AuNP-treated *P. frutescens* contains AuNPs in mesophyll cells, which implies the influx of AuNPs through the stomatal pathway. (**b**) Intensity variations over the A–A′ and B–B′ lines of (**a**). High intensity values (~ 60 × 10^3^) in the intensity profile indicate the existence of AuNPs along the B–B′ line for the AuNP-treated image. The A–A′ line for the control image does not show high intensity values. (**c**) Comparison of SEM images showing the mesophyll cells of the control and AuNP-treated *P. frutescens.* (**d**) Element analysis of particles adsorbed to mesophyll cells by using energy-dispersive X-ray spectroscopy (EDS). The absorbed particles are inferred as AuNPs because Au peaks are observed.

On the basis of the theoretical analyses on AuNPs passing through stomatal apertures, the absorbed AuNPs in the leaf of *P. frutescens* were experimentally observed by employing 3D X-ray micro-computed tomography (X-ray μCT; Fig. 4a). The leaf sample for X-ray imaging was the same as the leaf on which AuNP-laden drops were deposited. Following the fixation protocols for contrast-enhanced X-ray imaging, two *P. frutescens* models, namely, control and *P. frutescens* treated with AuNPs for 14 days (AuNP-treated), were prepared^46^. As a result, AuNPs were observed in mesophyll tissues of the AuNP-treated model, and none were observed in the control model. Higher light intensity values along the B–B′ line for the AuNP-treated model support the existence of AuNPs compared with smaller values along the A–A′ line for the control model (Fig. 4b). The accumulation of AuNPs in the mesophyll cells was also observed in SEM images (Fig. 4c). The result of energy dispersive X-ray spectroscopy (EDS) analysis verifies the existence of gold element in the absorbed AuNPs (Fig. 4d). These experimental results demonstrated that the absorbed AuNPs would diffuse from leaf stomata to stem tissues through interfacial cell regions. The possibility of AuNP translocation was identified by observing several cross-sections of vascular bundles (Fig. S3). In contrast to xylem vessels, particles accumulated on the walls of phloem vessels. The particles accumulated on the phloem walls might be the absorbed AuNPs^47^, but identifying the gold element accumulated on phloem walls by EDS analysis is impossible. While passing the biological barriers or membranes of plants, the surface of AuNPs absorbs additional organic coating, including proteins, lipids, and carbohydrates^14^. Identifying the element of particles may be difficult due to the organic coatings, but their effects are not completely revealed yet. These results support the translocation of AuNPs after diffusion of AuNPs via interfacial vacancies in mesophyll cells.

### Quantification of infiltrated AuNPs in P. frutescens

**Figure 5:**
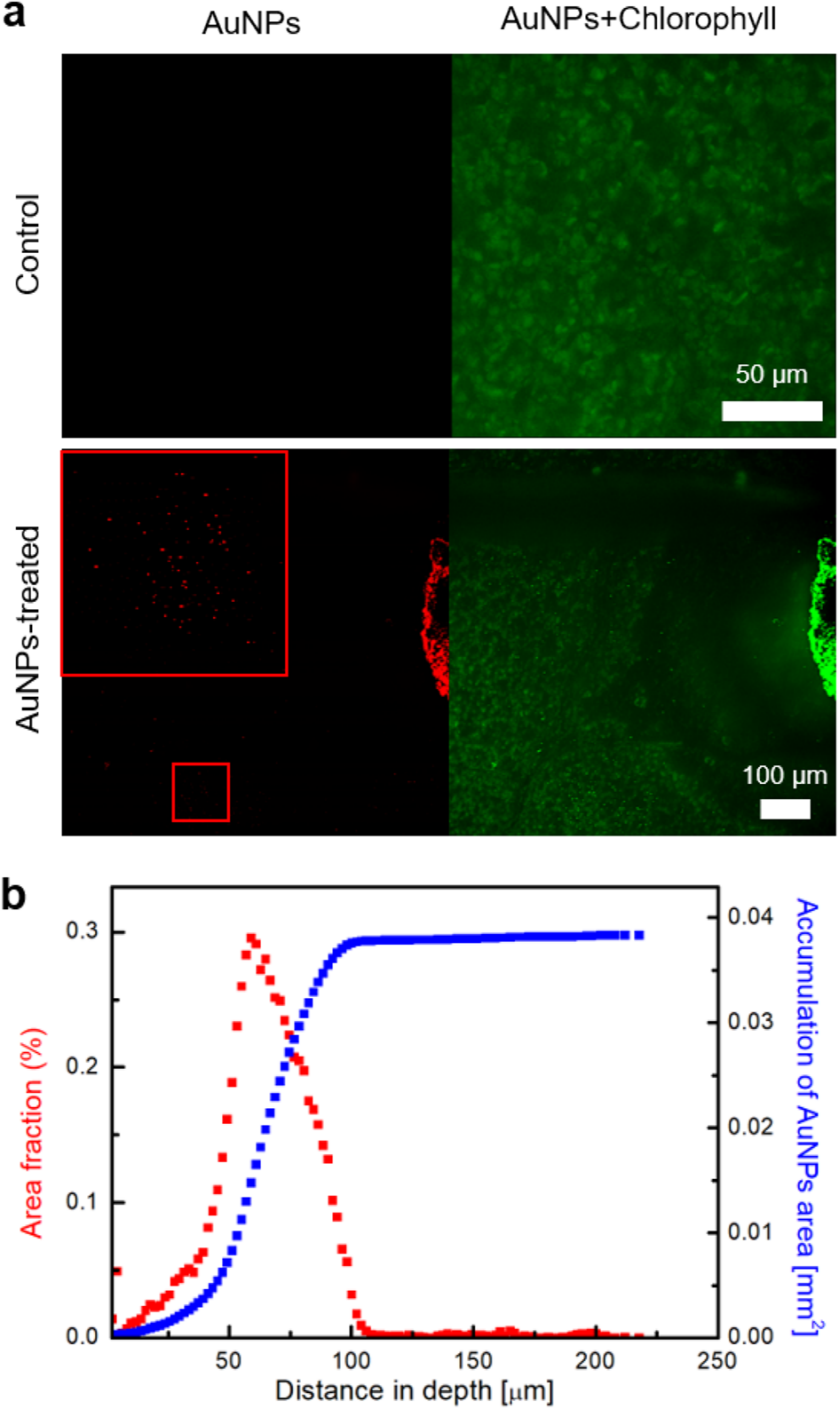
Spatial distributions of the infiltrated AuNPs in the region near the drop deposition point. (**a**) Projected images of the control and AuNP-treated *P. frutescence* leaves. The left images only show AuNPs, while the right images show AuNPs and chlorophylls. The edge indicates the region of drop deposition where saturation occurs. Inset in the red box shows a magnified image of the infiltrated AuNPs in plant tissues. (**b**) Variations in the area fraction (%) of AuNP fluorescence signals (red colors) and the accumulation of AuNP area (blue colors) along the depth direction.

To analyze the spatial distribution of absorbed AuNPs, we carried out TPEM analysis (Fig. 5). As a result, at the position on which drops of AuNP suspension were deposited, light intensity was saturated due to densely distributed AuNPs in the region near the CL of the drops. Thereafter, we explored the spatial distribution of AuNPs along the depth direction. Typical projected images are shown in Fig. 5a. For the control sample, the internal tissues of *P. frutescens* were observed at wavelengths where both AuNPs and chlorophylls were visible, while nothing was monitored at the wavelength where only AuNPs were visible. For the AuNP-treated sample, AuNPs were observed even ~ 700 µm away from the CL in the horizontal direction. Using the projections of AuNPs, the area fraction (*A*_*f*_) and accumulation area (*A*_*tot*_) of AuNPs were obtained along the depth distance (Fig. 5b). Here, the area fraction *A*_*f*_ is defined as the area ratio of AuNP fluorescence signals to the field of view for a projection, while the accumulation area *A*_*tot*_ is the sum of areas of AuNP fluorescence signals in the depth direction. As illustrated by red squares, the area fraction of AuNPs of about 3.8 × 10^–4^% was observed even at the region 200 µm away from the drop-deposited surface (see Supplementary Data for details). In other words, 292 AuNPs were distributed in the mesophyll cells when the area of fluorescence signals was assumed as clusters of AuNPs. Although most AuNPs were adsorbed by microstructures of the surface, such as trichomes and guard cells, we could identify the translocation of a few AuNPs into plant tissues by quantifying their 3D distribution.

### Translocation of AuNPs absorbed through the stomatal pathway

**Figure 6:**
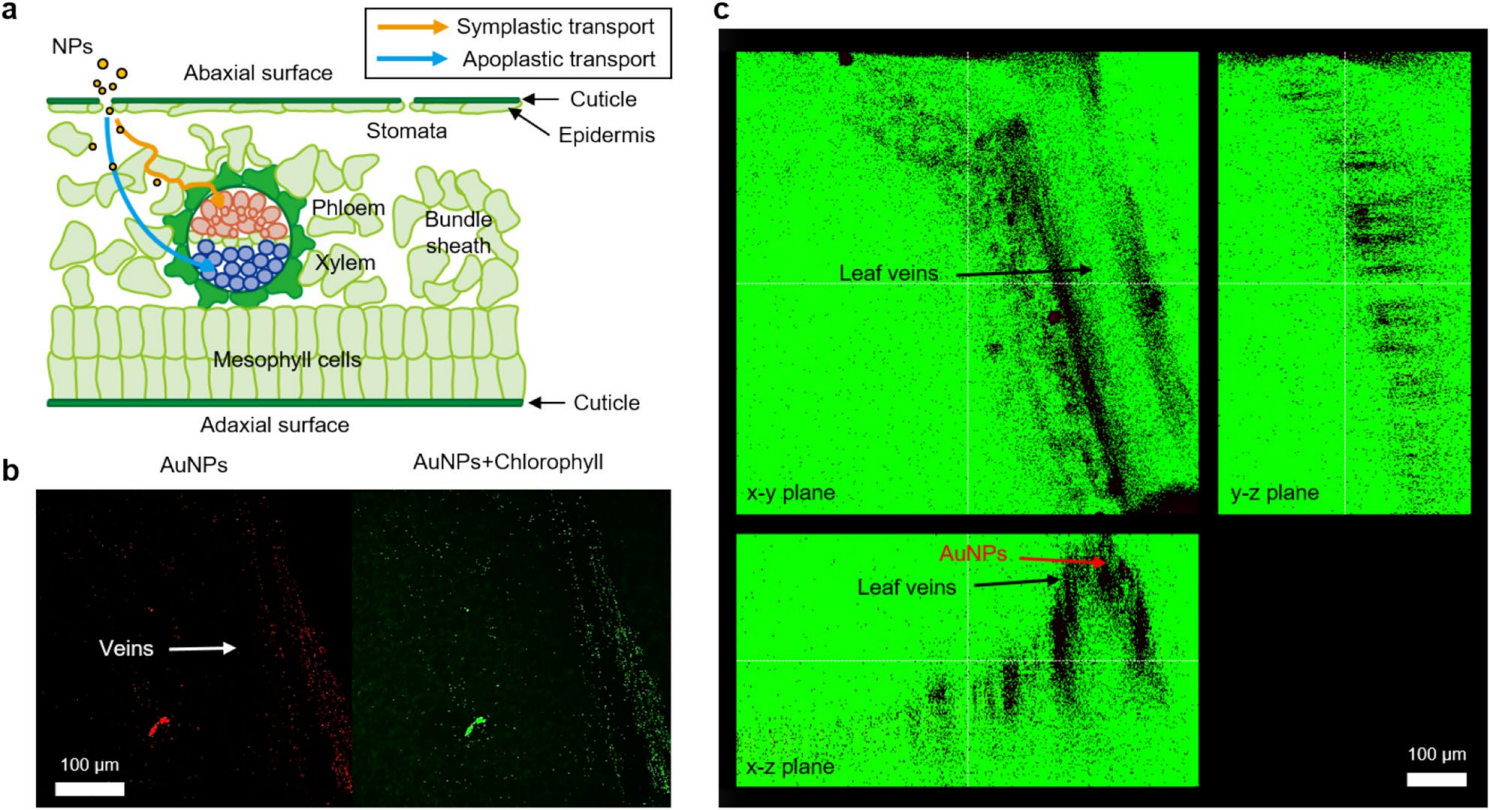
Translocation of the NPs absorbed through stomatal pathways. (**a**) Schematic showing the symplastic transport and apoplastic transport of NPs. (**b**) Accumulation of AuNPs near veins captured by TPEM imaging technique. Both images are projected in the depth direction to show the total distribution of AuNPs. White arrow indicates the position of leaf veins. (**c**) Color-filtered images showing the morphological features of leaf surface and 3D distribution of AuNPs. AuNPs are observed inside the veins, but they are not observed in the central region of vascular tissues.

In this study, we simulated the circumstance where ultrafine PM particle-laden drops are adsorbed to the *P. frutescens* surface and then infiltrated through stomatal pathways by using AuNP-laden sessile drops. The blockage of stomatal apertures by PM particles and the accumulation of NPs in the leaf and stem tissues would impair leaf photosynthesis and obstruct the metabolism of plants^21^. After entering the stomatal pores, PM particles would be diffused into mesophyll voids and translocated by different transport pathways: apoplastic or symplastic pathways^14^ (Fig. 6a). Apoplastic transport (transport in the intercellular space between cells) and symplastic transport (endocytosis and translocation in phloem vessels) have different size exclusion limits^48^. A previous study reported that the route for cellular translocation is influenced by the size of NPs^49^. Although the size exclusion limits for leaf uptake and translocation in vascular bundles are not completely understood, the accumulation of AuNPs along the axial direction of veins is observed by TPEM imaging (Fig. 6b). As illustrated in the x–z plane image of Fig. 6c, AuNPs were distributed along the walls of veins, but they were not observed in the center region of veins. From these results, we speculated that AuNPs arrived at mesophyll voids or bundle sheaths but did not reach vascular tissues due to the size exclusion limit of AuNPs.

## Conclusion

In this study, the absorption of AuNPs into *P. frutescens* via stomatal pathways was investigated by depositing AuNP-laden drops to explore the effect of ultrafine PM particles on edible plants. The experiment imitates the scenario wherein PM particle-laden drops remain on the abaxial surface of *P. frutescens*. Three dynamical behaviors in the AuNP-laden drops, namely, sedimentation, evaporation-driven convective flow, and shrinkage of drop interfaces, were analyzed to determine how these motions are related to AuNP adsorption. The sedimentation effect for AuNPs is relatively smaller than the internal convective flow effect, which gives rise to the accumulation of most AuNPs in the region near the CL. Sessile drops on the surface of *P. frutescens* are in Cassie state at the initial time, but they soon shift to a partially wetted state, that is, Wenzel state. During the evaporation process of AuNP-laden drops after becoming Wenzel state, the internal convective flow induces downward transport of AuNPs toward the CL. This internal convective flow allows AuNPs to be attached to the microstructures of leaves, such as stomata, trichomes, and grooves. In addition, the stick–slip behavior of the drop interface due to pinning and depinning of CL facilitates the accumulation of AuNPs at the CL and increases the local concentration of AuNPs at stomata located near the CL. This phenomenon would eventually lead to enhanced diffusion flux across the stomatal apertures and mesophyll voids. The amount of absorbed AuNPs through stomatal pathways was estimated using Fick’s law.

Contrast-enhanced X-ray imaging and EDS analysis showed the absorption of AuNPs into mesophyll voids. In addition, the spatial 3D distributions of the absorbed AuNPs were visualized by TPEM imaging, and the AuNPs in the depth direction were quantified from the obtained images. Related to the translocation of AuNPs, the TPEM results showed the existence of AuNPs in leaf veins of *P. frutescens* leaf. Although the exact transport route (apoplastic transport or symplastic transport) of AuNPs is difficult to distinguish, the 3D spatial distribution of the absorbed AuNPs was identified in this study. Compared with the present study on static sessile drops, natural circumstances might slightly alter the absorption of PM particle-laden drops. For instance, when a PM particle-laden drop impacts on the surface of a plant leaf, a vortex ring forms near the surface and the PM particles follow the vortex-induced flow and spread to other leaves^36^. Under high humidity, the time for drop evaporation increases^50^, followed by enhanced diffusion of NPs into the mesophyll voids.

The findings would provide the dynamic information on the inflow of ultrafine PM particles when patulous stomata are exposed to PM particle-laden drops. This study would be helpful for understanding the toxicity, physiological, and environmental effects of PM particles on plants, especially, edible plants and air-purifying plants, or even on a single plant cell scale^51^.

## Method

### Characterization of particles and stomata

*P. frutescens* was purchased from a local market and grown in an experimental room with constant temperature and humidity. AuNPs (100 nm in mean diameter; stabilized suspension in 0.1 mM PBS of pH ~ 7.4, reactant free) was purchased from Sigma Aldrich. Before conducting all the experiments, AuNP suspension was dispersed by ultrasonication for preventing agglomeration of AuNPs and improving the homogeneity and stability of the suspension. The sonication time was limited to three minutes, because too long a treatment duration longer than 5–10 min could rather cause formation of aggregates^52^. As an experimental comparison model of solid PM_2.5_ particles, standard Arizona test dusts (A1 dusts) were purchased from Powder Technology Incorporation. Macroscopic images of *P. frutescens* were captured by a digital camera (NIKON, d700). Detailed morphological features of particles and stomata were obtained by field emission SEM (JEOL JSM-7401F, JEOL) after coating them with platinum (SC7640 model, Quorum Technology, UK) for 30 s to avoid any charging effect.

### Experimental setup

The experimental setup for exposing *P. frutescens* to AuNPs is depicted in Fig. 1a. To remove all the impurities deposited on the leaf surface, *P. frutescens* sample was gently washed. Under solar irradiation for stomatal opening, several drops of AuNPs (the total volume was nearly constant as 30 μl) were smoothly deposited on the abaxial side of *P. frutescens* leaf for the absorption of AuNPs through the stomatal pathway.

### Contact angle measurement

Contact angle (CA) and contact line (CL) of a 2 μl drop of AuNP suspension were measured once by using Smartdrop (Femtofab, Korea). The drop was deposited on a slide glass or the abaxial side of *P. frutescens* surface. Temporal variations in CA and CL values were analyzed with software ImageJ.

### Measurement of zeta potential and hydrodynamic diameter

Zetasizer Nano ZS analyzer (Malvern Instruments, Worcestershire, UK) and a Perkin Elmer Lambda 900 UV/VIS/NIR Spectrometer, respectively, were used to measure the hydrodynamic particle size and zeta potential of AuNPs and dust particles to estimate the formation and/or aggregation of AuNPs and PM particles. The refractive index and absorbance of AuNPs were set as 0.2 and 3.32, respectively, at the wavelength of 633 nm. Given the absence of optical information of A1 dusts, the refractive index and absorbance of the A1 dusts were assumed to be 1.5 and 0, respectively. About 10 µl of AuNP suspension was diluted in a cuvette by using 4 ml of deionized water to satisfy the scale of nanomoles. The measurements were conducted ten times at 25 °C, and the results were statistically averaged.

### Contrast-enhanced X-ray imaging

Transport of AuNPs by convective internal flow in a sessile drop was observed at the 6C Biomedical imaging beamline of PAL (Pohang Accelerator laboratory, Korea). A monochromatic X-ray beam of 14 keV passes through the sample, and X-ray images were captured by a high-speed camera (PCO AG, Germany) located at 100 mm behind the sample. A microscope (Optique Peter, Lentilly, France) was attached in front of the camera. A drop of 100 nm AuNP suspension was deposited on a glass slide and the abaxial surface of *P. frutescens*. The spatial resolution was 0.165 µm, and the exposure time was 1000 ms.

### TPEM imaging

A commercial TPEM (TCS SP5 II MP, Leica) was used in this study. The microscope used a Ti–sapphire laser (Chameleon Vision II, Coherent) with specifications of 140 fs pulse width, 80 MHz repetition rate, and 20 × objective lens (HCX APO L 20 ×, 1.0NA water immersion, Leica, Germany). Laser power was measured by a power meter (S310C, Thorlabs). Excitation laser was tuned to 780 nm for both chlorophyll and AuNPs. Excitation laser power was approximately 60–100 mW depending on the imaging depth. Emission light was spectrally resolved by two NDD channels consisting of a set of dichroic mirrors of 560 and 620 nm. The acquired images were displayed in two pseudo-colors of *green* (630–680 nm) for chlorophyll and *red* (565–605 nm) for AuNPs. Two photon images consisted of 512 × 512 pixels, and the field of view was 775 μm × 775 μm. Image projection was performed by using Leica Application Suite software (LAS AF Lite, Leica). Post-processing of the acquired TPEM results was conducted for quantitative analysis of AuNPs by using ImageJ. The original images were transformed into 8-bit images, and a threshold function was applied. Using the converted binary images, we analyzed the number and area of AuNP signals in a slice and calculated the distribution of AuNPs along the depth direction.

### Particle imaging velocimetry (PIV) technique

Polystyrene particles of 1 µm in diameter were used as flow tracers. The density of the particles was 1.05 g/cm^3^, and the dynamic viscosity of the suspension was assumed as 10^–3^ Pa∙s. An inverted fluorescence microscope (Zeiss Axiovert 200, Zeiss, Germany) attached with an optical long-pass filter (λ > 550 nm) was utilized to observe tracer particles seeded in flow. A high-speed camera (PCO AG, Germany) was used to capture images of tracer particles with an exposure time of 500 ms. From the captured flow images, velocity fields of flow in a sessile drop placed on a flat glass surface were obtained via PIV. PIV view software (PIVTEC, Germany) was used to obtain the velocity field information of flow.

### Statistics

Error bars in all the graphs indicate standard deviations (SD) for values of three and over. Several SD were so small that they were hidden by data points.

### Ethics declarations

The authors declare that this experimental research on plants comply with local and national regulations and relevant permission has been obtained for the same.

Permission has been obtained.

## Supplementary Information

Supplementary Information 1.

Supplementary Information 2.

## Data Availability

All data required to evaluate the conclusions in the paper are present in the main text and/or the Supplementary Materials. Additional data related to this paper may be requested from the authors.
