# Supplementary material for: Adsorption of nanoparticles suspended in a drop on a leaf surface of Perilla frutescens and their infiltration through stomatal pathway
Source: Sci Rep. 2021 Jun 2;11:11556. doi: 10.1038/s41598-021-91073-x (PMC8172645; doi:10.1038/s41598-021-91073-x)
Supplement: Supplementary file 2 — Supplementary Information 2. [file 41598_2021_91073_MOESM2_ESM.docx]

**Supplementary Information**

**
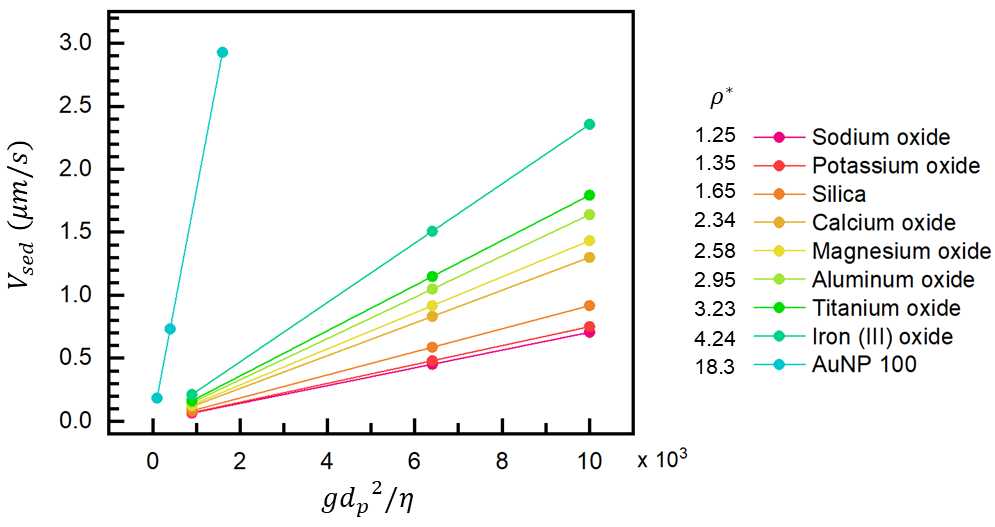
**

**Figure S1. Variations of theoretical sedimentation velocity of particles (*V_sed_*) according to dimensionless particle parameter (**${\boldsymbol{g}\boldsymbol{d}_{\boldsymbol{p}}}^{\boldsymbol{2}}\boldsymbol{/\eta}$**) and dimensionless density (**$\boldsymbol{\rho}^{\boldsymbol{*}}$**). *g*, *d_p_* and** $\boldsymbol{\eta}$ **indicate the gravitational acceleration, particle diameter and dynamic viscosity of suspension, respectively.** $\boldsymbol{\rho}^{\boldsymbol{*}}$ **=** $\mathbf{(}\boldsymbol{\rho}_{\boldsymbol{p}}\mathbf{/}\boldsymbol{\rho}_{\boldsymbol{f}}\mathbf{-1)}$**, where** $\boldsymbol{\rho}_{\boldsymbol{p}}$ **and** $\boldsymbol{\rho}_{\boldsymbol{f}}$ **are the densities of particles and suspension, and** $\boldsymbol{\rho}_{\boldsymbol{f}}$ **was assumed as 1 g/cm^3^. Circular points indicate *d_p_* of 0.1, 0.2, 0.3, 0.4, 0.8 and 1** $\text{μ}$**m from left to right. See details in Table S1.**

**
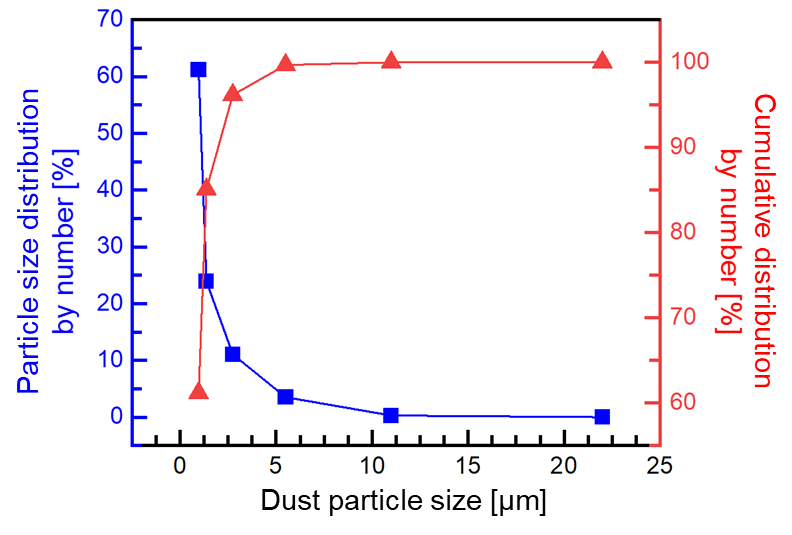
**

**Figure S2. Particle size distribution and cumulative distribution with respect to particle size of Arizona road dust (A1 dust). The data were converted by assuming them as spherical dust particles using the particle size distribution by volume provided in the website of Powder Technology Inc.**


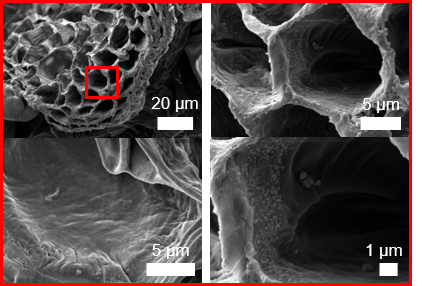


**Figure S3. SEM images showing the cross-sections of vascular bundles of AuNP-treated *P. frutescens.***

|  | Quantity in A1 dust [%] | Density [g/cm^3^] | Particle diameter [$\text{μ}$m] vs. Sedimentation velocity [$\text{μ}$m/s] | | Settling distance  [mm] (30 min) |
| --- | --- | --- | --- | --- | --- |
| AuNP | - | 19.3 | $d_{p}$ = 0.1 | 0.102 | 0.183 |
|  |  |  | $d_{p}$ = 0.2 | 0.407 | 0.732 |
|  |  |  | $d_{p}$ = 0.4 | 1.627 | 2.928 |
| Silica | 69-77 | 2.65 | $d_{p}$ = 0.3 | 0.083 | 0.149 |
|  |  |  | $d_{p}$ = 0.8 | 0.587 | 1.056 |
|  |  |  | $d_{p}$ = 1.0 | 0.917 | 1.650 |
| Aluminum oxide | 8-14 | 3.95 | $d_{p}$ = 0.3 | 0.148 | 0.266 |
|  |  |  | $d_{p}$ = 0.8 | 1.049 | 1.888 |
|  |  |  | $d_{p}$ = 1.0 | 1.639 | 2.950 |
| Calcium oxide | 2.5–5.5 | 3.34 | $d_{p}$ = 0.3 | 0.117 | 0.211 |
|  |  |  | $d_{p}$ = 0.8 | 0.832 | 1.498 |
|  |  |  | $d_{p}$ = 1.0 | 1.300 | 2.340 |
| Potassium oxide | 2-5 | 2.35 | $d_{p}$ = 0.3 | 0.068 | 0.122 |
|  |  |  | $d_{p}$ = 0.8 | 0.480 | 0.864 |
|  |  |  | $d_{p}$ = 1.0 | 0.750 | 1.350 |
| Sodium oxide | 1-4 | 2.27 | $d_{p}$ = 0.3 | 0.064 | 0.114 |
|  |  |  | $d_{p}$ = 0.8 | 0.452 | 0.813 |
|  |  |  | $d_{p}$ = 1.0 | 0.706 | 1.270 |
| Iron (III) oxide | 4-7 | 5.24 | $d_{p}$ = 0.3 | 0.212 | 0.382 |
|  |  |  | $d_{p}$ = 0.8 | 1.508 | 2.714 |
|  |  |  | $d_{p}$ = 1.0 | 2.356 | 4.240 |
| Magnesium oxide | 1-2 | 3.58 | $d_{p}$ = 0.3 | 0.129 | 0.232 |
|  |  |  | $d_{p}$ = 0.8 | 0.917 | 1.651 |
|  |  |  | $d_{p}$ = 1.0 | 1.433 | 2.580 |
| Titanium oxide | 0-1 | 4.23 | $d_{p}$ = 0.3 | 0.162 | 0.291 |
|  |  |  | $d_{p}$ = 0.8 | 1.148 | 2.067 |
|  |  |  | $d_{p}$ = 1.0 | 1.794 | 3.230 |

**Table S1. Comparison of AuNP and Arizona road dust (A1 dust) in density, theoretical sedimentation velocity and settling distance.**

|  | Particle type | Zeta potential [mV] | Average particle size [$\text{μ}$m] | Conditions |
| --- | --- | --- | --- | --- |
| [1] Lee et al. | Arizona road dust | -26.1 | - | measured in DI water at pH ~ 7 using Zetasizer |
| [2] Dong et al. | Dust in northwest China | -15 ~ -21 | 0.73 * |  |
| [3] Curtis et al. | Arizona road dust | - | 0.3 ** | Aerodynamic particle sizer |

* Value calculated using data of number size distribution. **Value calculated using Mie results.

**Table S2. Measured zeta potential and average particle size of road dust particles in previous studies.**

**References**

[1] Lee, J. *et al.* A study on the zeta potential measurement and the stability analysis of nano fluids using a particle image processing system. *Journal of ILASS-Korea* **8**, 16-22 (2003).

[2] Dong, F. Q. *et al.* Characterization of mineralogy and surface zeta potential of atmospheric dust fall in northwest China. *Miner Petrol* **109**, 387-395, doi:10.1007/s00710-014-0347-1 (2015).

[3] Curtis, D. B. *et al.* A laboratory investigation of light scattering from representative components of mineral dust aerosol at a wavelength of 550 nm. *J Geophys Res-Atmos* **113**, doi:Artn D0821010.1029/2007jd009387 (2008).
